# RACE AND ETHNIC DIFFERENCES IN ATTITUDES TOWARD FAMILY-BASED SUPPORT TO OLDER ADULTS IN THE U.S

**DOI:** 10.1093/geroni/igae098.3698

**Published:** 2024-12-31

**Authors:** Nekehia Quashie, Neil Mehta

**Affiliations:** University of Rhode Island, University of Rhode Island, Rhode Island, United States; The University of Texas Medical Branch, Galveston, Texas, United States

## Abstract

Prior research suggests cultural differences in norms of family responsibility to older adults and familial norms being more salient among minority families such as Black Americans relative to non-Hispanic Whites. However, there is little understanding of ethnic variations in attitudes toward familial responsibility for older adults among the Black American population. Using data drawn from the National Survey of American Life- Reinterview conducted between 2001 and 2003 and an analytic sample of African Americans (N=1,235), Caribbean Blacks (N=356), and non-Hispanic Whites (N=411) aged 40 and older, I examine race and ethnic differences in attitudes toward supporting older adults in need with finances, household chores, and personal care. Results show that for financial support to older adults, Caribbean Blacks were more likely than African Americans to support mixed family and government responsibility, relative to mainly familial responsibility. In contrast, non-Hispanic Whites were less likely than African Americans to support mainly government relative to mainly family responsibility to older adults. Regarding attitudes toward household support, Caribbean Blacks were more likely than African Americans to support mainly government responsibility, relative to family. There were no statistically significant race and ethnic differences in mixed or mainly government versus mainly family responsibility in supporting older adults with personal care needs. The findings underscore ethnic heterogeneity among Black Americans in attitudes toward the family being mainly responsible for some forms of support to older adults, which is increasingly important to inform culturally relevant policy initiatives to support vulnerable older adults and their families.

